# Targeted inactivation of EWSR1 : : FLI1 gene in Ewing sarcoma via CRISPR/Cas9 driven by an Ewing-specific GGAA promoter

**DOI:** 10.1038/s41417-025-00887-8

**Published:** 2025-03-15

**Authors:** Saint T. Cervera, Selene Martínez, María Iranzo-Martínez, Laura Notario, Raquel M. Melero-Fernández de Mera, Javier Alonso

**Affiliations:** 1 Unidad de Tumores Sólidos Infantiles. Instituto de Investigación de Enfermedades Raras. Instituto de Salud Carlos III. Majadahonda, Madrid, Spain; 2 Centro de Investigación Biomédica en Red de Enfermedades Raras. Instituto de Salud Carlos III (CB06/07/1009; CIBERER-ISCIII), Madrid, Spain; 3 Escuela de Doctorado. Universidad Autónoma de Madrid. Cantoblanco, Madrid, Spain; 4 Centro Nacional de Microbiología. Instituto de Salud Carlos III. Majadahonda, Madrid, Spain; 5 Facultad HM de Ciencias de la Salud. Universidad Camilo José Cela. Instituto de Investigación Sanitaria HM Hospitales, Madrid, Spain

**Keywords:** Targeted therapies, Paediatric cancer, Sarcoma, Sarcoma

## Abstract

We have recently demonstrated that genetic inactivation of EWSR1 : : FLI1 by CRISPR/Cas9, successfully blocks cell proliferation in a cell model of Ewing sarcoma. However, CRISPR/Cas9-mediated gene editing can exhibit off-target effects, and thus, precise regulation of Cas9 expression in target cells is essential to develop gene-editing strategies to inactivate EWSR1 : : FLI1 in Ewing sarcoma cells. In this study, we demonstrate that Cas9 can be specifically expressed in Ewing sarcoma cells when located downstream a promoter consisting of GGAA repeats and a consensus TATA box (GGAAprom). Under these conditions, Cas9 is selectively expressed in Ewing sarcoma cells that express EWSR1 : : FLI1 oncoproteins, but not in cells expressing wild-type FLI1. Consequently, Ewing sarcoma cells infected with GGAAprom>Cas9 and a specific gRNA designed to inactivate EWSR1 : : FLI1, showed successful EWSR1 : : FLI1 inactivation and the subsequent blockade of cell proliferation. Notably, GGAAprom>Cas9 can be efficiently delivered to Ewing sarcoma cells via adenoviral vectors both in vitro and in vivo, highlighting the potential of this approach for Ewing sarcoma treatment. Our results demonstrate that the CRISPR/Cas9 machinery is safe and specific for Ewing sarcoma cells when driven under a GGAAprom, paving the way for the development of cancer gene therapies based on the selective expression of genes with therapeutic potential.

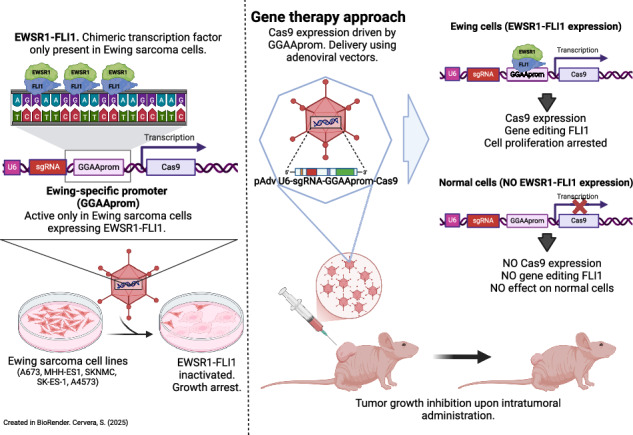

## Introduction

Ewing sarcoma is a devastating disease that primarily affects the bones of children and young adults. Recently, international collaborations have established a gold standard for the treatment of these patients based on standard chemotherapy/radiotherapy, and surgery, enabling a 3-year overall survival rate of 82% [1, 2]. However, the prognosis for patients with metastatic disease, localized disease at the axial skeleton (for which surgery is not a suitable option), or those who are refractory to first-line treatments remains particularly dramatic, as second-line treatments fail to offer a meaningful life expectancy for these patients [3]. Therefore, it is absolutely necessary to develop new disruptive therapies that truly represent a breakthrough in the treatment of this disease [4, 5].

Since conventional treatments with chemotherapy and radiotherapy have probably reached their maximum potential to cure these patients, identifying molecular targets that allow the development of Ewing-specific therapies is crucial. Among all the possible molecular targets, the fusion oncoprotein EWSR1 : : FLI1, a chimeric transcription factor that is characteristic and exclusive to Ewing sarcoma, clearly stands out. EWSR1 : : FLI1 is a fusion protein generated from a reciprocal chromosomal translocation (i.e., t (11;22)), in which the N-terminal region of the *EWSR1* gene is fused to the C-terminal region of the transcription factor *FLI1*, which contains the DNA binding domain [6]. In some cases (<15% of Ewing sarcoma tumors), other reciprocal translocations are generated, fusing *EWSR1* to other transcription factors of the ETS family of transcription factors (i.e., *ERG and FEV*), giving rise to functionally equivalent chimeric transcription factors [7].

Over the last 20 years, it has been clearly established that EWSR1 : : FLI1 plays a central role in Ewing sarcoma pathogenesis. EWSR1 : : FLI1 regulates the expression, directly or indirectly, of hundreds of genes that, taken together, are capable of promoting cell proliferation and blocking cell differentiation [8–10]. EWSR1 : : FLI1 also alters the epigenetic program in Ewing sarcoma cells, further contributing to the oncogenic profile [11–13]. In addition to these molecular findings, there is also functional evidence that clearly reveals the key role of EWSR1 : : FLI1 in the pathogenesis of Ewing sarcoma. Thus, dozens of studies have demonstrated that EWSR1 : : FLI1 knockdown, using for example RNA interference approaches, impairs the proliferation of Ewing sarcoma cells both in vitro and in vivo [9, 14, 15].

In light of these molecular and functional data, it is widely accepted that these chimeric transcription factors are the main oncogenic drivers in Ewing sarcoma [7]; thus, targeted inactivation of EWSR1 : : FLI1 should be the most effective therapy for Ewing sarcoma [16].

We have recently shown that *EWSR1 :* *: FLI1* gene inactivation via a CRISPR/Cas9 approach efficiently inactivates the *EWSR1 :* *: FLI1* gene in A673 Ewing sarcoma cells, blocks cell proliferation, and induces cell senescence, suggesting that *EWSR1 :* *: FLI1* gene inactivation via a gene therapy approach is feasible and could be a new therapeutic strategy for Ewing sarcoma treatment [17]. However, CRISPR/Cas9 technologies must be used with exquisite specificity before they can be applied clinically [18–20]. One way to improve specificity could include designing a molecular strategy in which the Cas9 nuclease is expressed exclusively and specifically in Ewing sarcoma cells.

Interestingly, EWSR1 : : FLI1 has a unique property that could be exploited therapeutically. In fact, EWSR1 : : FLI1 binds GGAA microsatellites formed by the concatenation of dozens of GGAA motifs and activates the expression of genes located near these DNA elements [7, 9]. This characteristic is exclusive to EWSR1 : : FLI1 and to our knowledge, no other transcription factors have been shown to have similar characteristics. Interestingly, native FLI1, which shares the same DNA-binding domain as EWSR1 : : FLI1, is not able to transactivate these GGAA repeat sequences.

In this work, we show that a minimal GGAA-based promoter, which contains concatemerized GGAA repeats and a TATA-box (GGAAprom), is able to drive high Ewing-specific expression of a gene of interest. Using Cas9 and specific sgRNAs designed to inactivate EWSR1 : : FLI1 as a therapeutic approach, we demonstrated that this strategy is effective in vitro when lentiviral and adenoviral vectors are used as delivery strategies. In addition, the use of adenovirus in vivo revealed that this strategy allows the expression of luciferase only in tumor cells but not in normal tissues, indicating that this strategy is safe. Finally, we demonstrated that adenovirus harboring Cas9 under the regulation of the GGAA promoter were able to reduce tumor growth in vivo. Our results justify additional preclinical research to advance in the development of this new gene therapy for Ewing sarcoma.

## Materials and methods

### Cell lines

The Ewing sarcoma cell line A673 (CRL-1598), the fibrosarcoma cell line HT1080 (CCL-121), the osteosarcoma cell lines U2-OS (HTB-96) and Saos-2 (HTB-85) and the HEK293T cells (CRL-3216); were purchased from the American Type Culture Collection (Manassas, VA, USA). MHH-ES1 (ACC167) and SK-N-MC (ACC203) were purchased from the DSMZ-German Collection of microorganisms and cell cultures (Braunschweig, Germany). The Ewing sarcoma cell lines A4573 were generously given by Dr. S. Navarro (University of Valencia, Spain). A673, SK-N-MC, HEK293T and HT1080 cell lines were maintained in DMEM; A4573, MHH-ES1 and U2-OS were maintained in RPMI 1640; Saos-2 cell line was maintained in McCoy’s medium. All media were supplemented with 10% fetal bovine serum, 50 U/mL penicillin and 50 µg/mL streptomycin (Corning, Glendale, AZ, USA). Cell lines were checked by STR profiling and periodically tested for mycoplasma contamination (Mycoalert mycoplasma detection kit, Lonza, Rockland, ME, USA).

### Cloning of GGAAprom sequence

The GGAA-microsatellite located in the NR0B1(DAX1) gene promoter ([9]) was amplified by PCR with primers DAX1-PF1 5’-GGGGGTACCTCTCACAGGCAGAATGAAAT-3’ and DAX1-PR 5’-TACCAGCTGATACAGAATCATT-3’ from genomic DNA of the Ewing sarcoma cell line A4573. The PCR product was purified and then cloned into pGEM-T Easy Vector (Promega Corp., Fitchburg, WI, USA) generating a consensus TATA-box during cloning. Subsequently, GGAAprom fragment was subcloned by enzyme restriction and ligation into the pGL3-Basic luciferase reporter vector (Promega Corp.).

### Luciferase reporter assays

HEK293T cells were transfected with the reporter plasmids pGL3-Basic or pGL3-GGAAprom, the expression plasmids pCI-EWS/FLI1 or pCI-FLI1 described previously [9, 21]) and the control reporter plasmid pRL (Promega Corp.) Firefly luciferase activity was measured with the Dual-Glo® Luciferase Assay System (Promega Corp.) and normalized to *Renilla* luciferase activity as a control for transfection efficiency.

### Establishment of cell lines expressing LUC2 under the control of GGAA promoters and luciferase assays

Lentiviral plasmids encoding the luciferase protein LUC2 downstream different promoters (GGAAprom, TATAless, GGAAless and GGAAless/TATAless) were produced by Vector Builder (Chicago, IL, USA). In addition to promoter>LUC2 cassettes, lentiviral plasmids include an EGFP:T2A:Bsd cassette under a CMV promoter for cell selection and monitoring of transfection. A673, MHH-ES1 and HT1080 cells were infected with lentiviral particles at two different MOI (0.5 and 5) and selected with blasticidin (50 µg/mL). To confirm similar levels of lentiviral transduction, the percentage and intensity of GFP positive cells were quantified by flow cytometry with a MACS Quant Analyser flow cytometer (Miltenyi Biotec, Cologne, Germany) and FlowJo™ software (v.10.7.1, De Novo Software, Pasadena, CA, USA). To quantify luciferase activity, stable cell lines were seeded in triplicate in a 6-well plate at a density of 200,000 cells per well and cultured for 72 h. Then, cells were washed twice with PBS 1x and lysed in 500 µl of passive lysis buffer (PLB) (Promega Corp.). Protein concentration was determined with a Pierce microBCA™ protein assay kit (Thermo Fisher Scientific, Waltham, MA, USA). To avoid interference between the BCA reagents and PLB, samples were diluted 10-fold, and the standard curve was prepared in PLB 0.1x. After that, 100 ng of total protein was used to measure the luciferase activity with the Luciferase Assay Reagent (LAR) (Promega Corp.) according to the manufacturer’s instructions. Luciferase activity was measured in an Infinite M200 microplate reader (Tecan, Männedorf, Switzerland).

### Establishment of cells expressing Cas9 under the control of GGAA promoters and analysis of the effect of EWSR1 : : FLI1 gene editing on cell proliferation

A673, MHH-ES1 and HT1080 cells were infected with lentiviral particles (MOI = 5) encoding Cas9 under the control of GGAAprom (cell lines GGAAprom>Cas9) or GGAAless (cell lines GGAAless>Cas9) promoters. HT10180 cells were also infected with lentiviral particles harboring Cas9 under the control of the constitutive promoter EFS (EFS>Cas9), a short version of the full-length EF1A promoter. After selection with blasticidin (50 µg/mL), cells were sorted by flow cytometry to obtain a cell population with high levels of GFP expression. Cas9 expression levels were confirmed by western-blot with an anti-Cas9-specific antibody (#Ab191468, Abcam, Cambridge, UK).

To analyze the effect of EWSR1 : : FLI1 editing on cell proliferation, stable cell lines transduced with GGAAprom>Cas9, GGAAless>Cas9, or EFS>Cas9 were infected with lentiviral particles (MOI = 5) harboring lentiviral expression vectors of gRNAs directed against FLI1 exon 2 or FLI1 exon 9 as previously described [17]. After infection, cells were briefly selected with hygromycin (350 µg/mL) during 4 days and then seeded in replicate (1 × 10^3^ cells per well) in 96-well black plates. Cell proliferation was determined at 0, 4 and 7 days using a resazurin metabolic assay. At the indicated times, 20 µL of resazurin (0.15 mg/mL) (#R7017, Sigma Aldrich, Saint Louis, MO, USA) were added to cells and incubated for 5 h at 37 °C. Fluorescence was measured at 560 nm excitation/590 nm emission filter set in an Infinite M200 microplate reader (Tecan).

### Western-blot

Total protein was isolated from cell pellets lysed in RIPA buffer (1x PBS, 0.1% SDS, 1% NP-40, 0.5% sodium deoxycholate) supplemented with EDTA 1 mM and a protease and phosphatase inhibitor cocktail (Roche, Basel, Switzerland). Protein concentration was determined with the Pierce BCA™ protein assay kit (Thermo Fisher Scientific). For Western blotting, 10–30 µg of total protein extracts were subjected to electrophoresis on 4–15% polyacrylamide precast gels (Bio-Rad, Hercules, CA, USA) and blotted onto 0.2 µm PVDF membranes (Trans-Blot Turbo Mini, Bio-Rad). The membranes were blocked with 5% skim milk powder in 1x Tween-Tris-buffered saline (TTBS) and subsequently incubated with the corresponding primary antibodies diluted in the same buffer overnight at 4 °C. The next day, the membranes were washed in 1x TTBS, incubated with the secondary HRP-conjugated antibody, and washed again. The membranes were finally incubated with enhanced chemiluminescence (ECL) reagents (Merck Millipore, Darmstadt, Germany) and photographed on an Amersham ImageQuant™ 800 (Cytiva). The membranes were incubated sequentially with different antibodies after stripping for 30 min at 65 °C with stripping buffer (Tris-HCl, pH 6.8; SDS, 10%; β-mercaptoethanol, 0.7%). The primary antibodies used were as follows: anti-FLI1, 1:500 (#133485, Abcam); anti-DAX1 (NR0B1), 1:1 000 (2F4 clone) (a generous gift of Dr. E. Lalli; [22]); anti-CD44, 1:1 000 (#ab51037, Abcam); and anti-β tubulin, HRP-conjugated, 1:10 000 (#ab185057, Abcam). Anti-mouse m-IgG-HRP (#sc-516102) and anti-rabbit IgG-HRP (#sc-2357) secondary antibodies were purchased from Santa Cruz Biotechnology (Dallas, TX, USA).

### Analysis of gene editing

Genomic DNA was isolated from previously frozen cell pellets using the QIAamp DNA Mini Kit (Qiagen, Hilden, Germany). DNA fragments covering the gRNA target were amplified by PCR using the primers described in Supplementary Fig. S6. PCR amplicons were subsequently sequenced by Sanger sequencing and electropherograms were analyzed with the ICE CRISPR analysis tool (Synthego, Redwood City, CA, USA) to quantify the percentage of indel mutations.

Gene editing was also confirmed by T7 endonuclease assay. Briefly, PCR products obtained from genomic DNA were purified using the QIAquick PCR Purification Kit (Qiagen), and then 200 ng of the purified DNA was denatured for 5 min at 95 °C and afterwards allowed to align slowly using a first step consisting of a − 2 °C/s ramp rate from 95 to 85 °C and a second step consisting of a − 0.1 °C/s ramp rate from 85 to 25 °C. Next, 1 µL (10,000 UI/mL) of T7 Endonuclease I (#M0302, New England Biolabs, Ipswich, MA, USA) were added to the re-annealed PCR products and incubated at 37 °C for 15 min. Finally, T7 endonuclease reaction was stopped with 1.5 mM EDTA on ice. The fragmented PCR products were analyzed by agarose electrophoresis, photographed, and the percentage of nuclease-specific cleavage products quantified by densitometry (Fiji software).

Gene editing in tumor tissues was analyzed by ultra-deep next generation sequencing. Briefly, PCR amplicons were purified with Sera-Mag Select beads (Cytiva, Marlborough, MA, USA), PCR-indexed, repurified, quantified, and pooled. Sequencing was performed in a MiSeq sequencer (Illumina, San Diego, CA, USA) using a 2×250 pb paired-end reads scheme. Percentage of edited reads was calculated with CRISPResso2 software [23].

### In vitro analysis of adenovirus infection: reporter assays

Adenoviral vectors containing an hPGK>mCherry cassette and the GGAAprom>LUC2 (pAd-hPGK>mCherry-GGAAprom>LUC2) or EF1A > LUC (pAd-hPGK>mCherry-EF1A > LUC2) cassettes were synthesized by Vector Builder. Adenovirus were produced by the Viral Production Unit (VPU) of the Universitat Autonoma de Barcelona (UAB) (Barcelona, Spain). Cells were seeded in triplicate in 96-well plates (3000 cells per well) and infected the next day with the aforementioned adenovirus at different MOI (25, 100, 400). Then, 48 h later, cells were washed with 1× PBS and lysed in 50 µL of 1x passive lysis buffer (PLB) (Promega) at room temperature for 15 min with agitation. Total protein and luciferase activity were measured following the protocol described above. In addition, cells were seeded in 6-well plates and treated similarly with adenovirus to quantify mCherry expression by flow cytometry (BD LSRFortessa™ X-20 Cell Analyser, Becton Dickinson, Franklin Lakes, NJ, USA) as a control for viral transduction.

### In vitro analysis of adenovirus infection: Cas9 therapeutic gene

Adenoviral vectors containing a reporter cassette (hPGK>mCherry), a gRNA expression cassette (U6 > FLI1_EX2 or U6 > FLI1_EX9), and a Cas9 expression cassette (GGAAprom>Cas9) were synthesized by Vector Builder. Adenovirus were produced by the Viral Production Unit (VPU) of the Universitat Autonoma de Barcelona (UAB). Cells were seeded in 6-well plates (5000 cells per well) and the next day, infected with adenovirus at a MOI = 25. Then, 48 h later, the medium containing the adenovirus was removed, and a fresh culture medium was added. Afterwards, cells in replicate wells were trypsinized and counted using trypan blue solution and an EVE™ automatic cell counter (NanoEnTek Inc., Seoul, Korea) at 3, 6 and 9 days. The remanent cells were centrifuged, and the pellets stored for protein and DNA extraction for western-blot and gene editing analysis respectively.

### In vivo experiments

All experiments in animals were performed in 8 to 10-week-old athymic female nude mice (CR ATH HO strain, Charles River, Les Oncins, France) and conformed to the Animal Welfare guidelines and protocols approved by the Animal Experimentation Ethics Committee of the Instituto de Salud Carlos III (CBA 25_2020 and CBA 24_2022) and Agriculture Department of the Madrid Region (PROEX 253.8/20 and PROEX 302.3/22).

For in vivo biodistribution experiments, mice were injected intravenously with 10^10^ viral particles of adenovirus (pAd-hPGK>mCherry-GGAAprom>LUC2 or pAd-hPGK>mCherry-EF1A > LUC2) in 100 µL PBS. After 72 hours, animals were inoculated i.p. with 100 μl of Xenolight™ Potassium Salt Bioluminescent substrate (15 mg/ml) (Perkin Elmer, Waltham, MA, USA) and photographed in an IVIS Lumina XRMS (PerkinElmer). Images were acquired in automatic model and analyzed with the Living Image 4.8 software (Perkin Elmer). Afterward, animals were euthanized and representative fragments of 12 different organs (spleen, liver, bone, lung, muscle, uterus, ovary, large intestine, heart, small intestine, kidney, and brain) were frozen and stored at -80 °C until they were processed to quantify the luciferase activity in tissue protein extracts. Briefly, tissues were weighted and lysed in 1x passive lysis buffer (Promega) (750 µL lysis buffer/100 mg tissue) in tubes with ceramic beads (# CK-28R; Bertin Technologies, Montigny-le-Bretonneux, France) in a Precellys Evolution tissue homogenizer (Bertin Technologies) (6,800 rpm, 3 cycles 20 seg each, 30 seg pause between cycles). Protein quantification and luciferase assays were performed as described above. For in vivo studies in tumors, mice were inoculated subcutaneously with 5 × 10^6^ A673 cells and when tumors reached a volume of 250 mm^3^, tumors were injected intratumorally with a unique dose of 10^9^ viral particles in 100 µL PBS. Luciferase activity, at 3- and 6-days post intratumoral inoculation, was determined by IVIS imaging in whole animals as described above. At the end of the experiment, tumors were excised and luciferase activity in tumor lysates was quantified as above.

For efficacy studies of therapeutic adenovirus (those expressing Cas9 protein under the GGAAprom), mice were inoculated subcutaneously with 5 × 10^6^ A673 and when tumors reached a determined volume, animals were randomly distributed into two groups (PBS and treated group). To distribute the animals equally between both groups and avoid possible biases, the animals were first ordered from largest to smallest tumor volume, and then randomly distributed to one group or the other. Animals were treated with three intratumoral injections at 48–72 h intervals. Two independent experiments using two different doses of adenovirus were performed (1 × 10^9^ VP/dose and 5 × 10^10^ VP/dose). Tumor growth was monitored each 2-3 days with a caliper and tumor volume was calculated as: ((length × width^2^) × π)/6.

### Immunohistochemistry Ki67

Tissue sections (3 µm) from paraffin embedded tumors were deparaffinized, treated with HIER2 buffer for antigen retrieval (Leica Biosystems, Nussloch, Germany) and stained with Ki67 antibody (clone SP6, 1/500 dilution, # RM-9106, Thermo Scientific, Kalamazoo, MI, USA). After DAB development, samples were counterstained with hematoxylin. All steps were performed in a Bond RX automatic stainer (Leica Biosystems). Stained tissues were scanned at 40x in a NanoZoomer-SQ digital slide scanner (Hamamatsu, Shizuoka, Japan). Percentage of Ki67 positive cells was determined using QuPath software (v0.5.1) [24].

### Statistical analysis

The number of independent experiments performed for each experiment is indicated in the corresponding figure legend. For animals, a minimum of five animals per group were used and the exact number used per group is also indicated in the corresponding figure legend. Statistically differences between means were calculated with one-way or two-way ANOVA or Student’s *T*-test as appropriate. All statistical tests were two-sided and adjusted for multiple comparisons as appropriate. Statistical analysis were carried out with GraphPad software, version 9. *P* values < 0.05 were considered statistically significant.

## Results

EWSR1 : : FLI1 have the unique ability to activate transcription by binding GGAA microsatellites in or near gene target promoters. Keeping this unique feature in mind, we reasoned that a therapeutic gene placed downstream of a GGAA repeat sequence would be expressed exclusively in Ewing sarcoma cells, making this gene therapy strategy highly specific. To confirm this, we first cloned the GGAA repeat sequence present in the NR0B1 gene [9] (henceforth GGAAprom) into a luciferase reporter vector (pGL3). We selected the GGAA microsatellite present in the NR0B1 (also called DAX1) promoter of Ewing sarcoma A4573 cells because it was associated with high NR0B1 gene expression in previous studies [9], suggesting that this microsatellite is highly effective at promoting the expression of a putative therapeutic gene. During the cloning of this GGAA microsatellite in the reporter vector, we also added a TATA-box sequence downstream of the GGAA repeat region (DNA sequences are available in Supplementary Fig. S1).

**Fig. 1:**
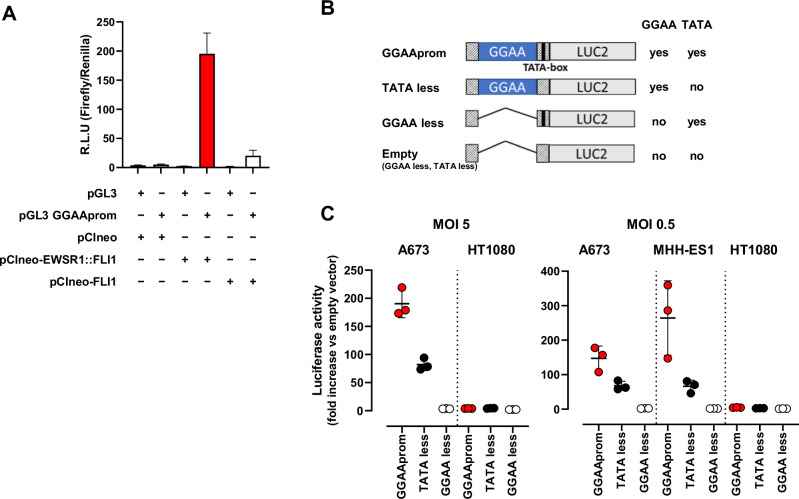
Specific activation of a promoter constituted by a GGAA repeat region and a TATA-box (GGAAprom) by EWSR1 : : FLI1. **A** In vitro reporter assay. HEK293T cells were co-transfected with the reporter vector pGL3 GGAAprom and the expression vectors pCIneo-EWSR1-FLI1 or pCIneo-FLI1. After 48 h, firefly luciferase activity was quantified and normalized to renilla luciferase activity. EWSR1 : : FLI1 induced specifically the expression of the reporter gene luciferase (mean ± SD of one experiment done by triplicate). **B** Schematic representation of the different promoters used for lentiviral infections, indicating the presence or absence of GGAA repeat region and TATA-box. **C** A673 and MHH-ES1 Ewing sarcoma cells and HT1080 fibrosarcoma cells were infected at two different MOI with lentivirus harboring the luciferase reporter gene under the control of the promoters described in B). Luciferase activity in stably infected cells was quantified and normalized to cells infected with an empty vector. High luciferase activity was observed in Ewing sarcoma cells infected with GGAAprom including the GGAA repeats and the TATA-box. Luciferase expression was not observed in HT1080 infected with any construct (mean ± SD of three experiments).

Next, pGL3-GGAAprom was transfected into 293 T cells in the presence of an EWSR1 : : FLI1 or FLI1 expression vector, and the activity of the luciferase reporter gene was analyzed. As shown in Fig. 1A, EWSR1 : : FLI1 was able to increase luciferase activity nearly 200-fold compared to the basal background, whereas native FLI1 increased luciferase activity less than 20-fold compared to the background. These results indicate that EWSR1 : : FLI1 is able to strongly activate GGAAprom and that EWSR1:FLI1 is significantly more efficient than native FLI1.

To study the specificity of GGAAprom and analyse the individual contributions of the GGAA repeat region and the TATA box to EWSR1 : : FLI1-mediated promoter activation, we generated stable cell lines via lentiviral infection. In these experiments, we used four different lentiviral vectors with different promoter sequences upstream of a luciferase reporter gene (Fig. 1B, supplementary Fig. S2): i) GGAAprom (including the GGAA-rich region and downstream TATA-box); ii) TATAless, a version of GGAAprom in which the TATA-box has been deleted but maintains the GGAA-rich region; iii) GGAAless, a version of GGAAprom in which the GGAA-rich region has been deleted but maintains the TATA-box; and iv) an empty vector in which both the GGAA-rich region and the TATA-box have been deleted. The Ewing sarcoma cell line A673 (expressing EWSR1 : : FLI1 type I protein but not native FLI1 protein) and the fibrosarcoma cell line HT1080, which express high levels of native FLI1 protein (Supplementary Fig. S3), were infected at MOI = 5, and luciferase activity was analyzed. As shown in Fig. 1C, A673 cells stably transduced with GGAAprom expressed high levels of luciferase. Interestingly, luciferase levels were significantly lower in A673 cells transduced with the TATA-less promoter than in those transduced with GGAAprom, indicating that a functional TATA box seems to be necessary to achieve maximal activation of GGAA-rich promoters by EWSR : : FLI1. Notably, no luciferase expression was detected in A673 cells transduced with GGAAless promoter, confirming that the GGAA-rich region is necessary to induce gene expression by the EWSR1 : : FLI1 fusion protein in Ewing sarcoma cells. Remarkably, no luciferase expression was detected in HT1080 cells with any of the vectors, confirming that GGAA-rich promoters cannot be activated by native FLI1 in cells, such as HT1080 cells, which express high levels of native FLI1 protein. We repeated these experiments using a MOI = 0.5, which ensures the integration of no more than one gene copy by cell. We also included one additional Ewing sarcoma cell line, MHH-ES1, which harbors an EWSR1 : : FLI1 type 2 fusion, covering the two more frequent gene fusions found in Ewing sarcoma (Fig. 1C).

As expected, luciferase activity was slightly lower in A673 cells infected at an MOI of 0.5 than in the same cells infected at an MOI of 5 with GGAAprom. Interestingly, luciferase activity was even greater in MHH-ES1 cells than in A673 cells infected with GGAAprom. Thus, GGAAprom, which contains a GGAA-rich region and a linked, functionally active, TATA box, can be efficiently activated by EWSR1 : : FLI1 in A673 and MHH-ES1 Ewing sarcoma cells but not in HT1080 cells, which express high levels of the native FLI1 protein. This characteristic makes GGAAprom particularly suitable as a specific promoter for gene therapy in Ewing sarcoma because of its high specificity for EWSR1 : : FLI1.

**Fig. 2:**
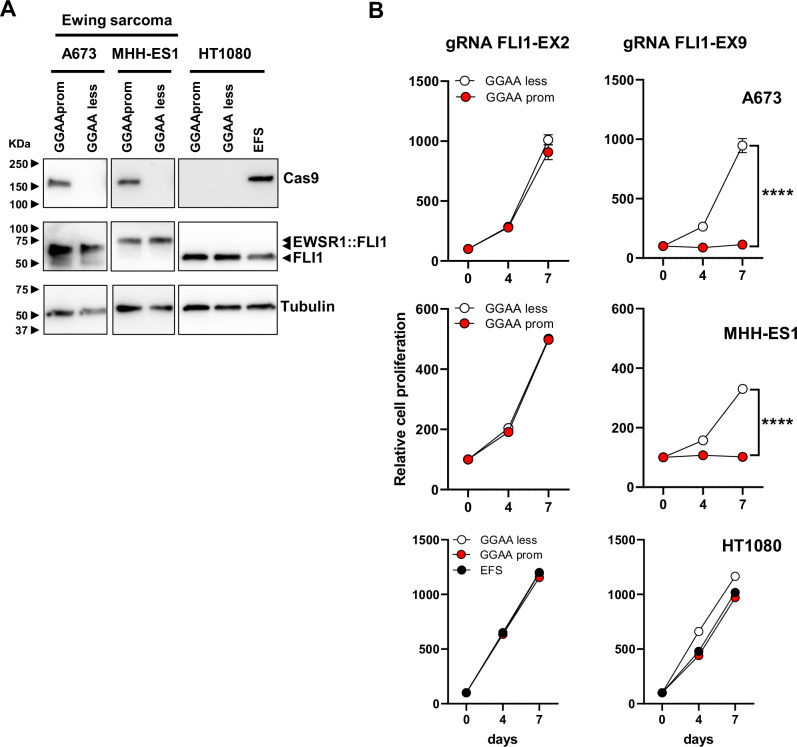
Expression of Cas9 in Ewing sarcoma cells blocks cell proliferation when combined with a FLI1 exon 9 gRNA. **A** Cas9 expression in A673 and MHH-ES1 Ewing sarcoma cells and HT1080 fibrosarcoma cells stably transduced with GGAAprom>Cas9, GGAAless>Cas9 and EFS>Cas9 lentivirus. High levels of Cas9 expression were specifically achieved in Ewing sarcoma cells infected with GGAAprom>Cas9. **B** Effect of gRNA FLI-EX2 and FLI1-EX9 on cell proliferation. Stably transduced cells were additionally infected with lentivirus harboring a gRNA against FLI1 exon 2 (negative control) or a gRNA against FLI1 exon 9, which causes the genetic inactivation of EWSR1 : : FLI1. The effect on cell proliferation was monitored for 7 days. Cas9 expression combined with gRNA FLI1-EX9 blocked cell proliferation only in Ewing sarcoma cells (mean ± SD of a representative experiment from a total of three; ****P < 0.0001, Student’s T-test).

As a proof of concept, we designed new lentiviral vectors in which the Cas9 nuclease was placed under the control of GGAAprom (GGAAprom>Cas9) or GGAAless promoter (GGAAless>Cas9). As shown in Fig. 2A, Cas9 was expressed in A673 and MHH-ES1 Ewing sarcoma cells infected with GGAAprom>Cas9 lentivirus but not in cells infected with GGAAless>Cas9 lentivirus. This result corroborates the findings obtained when using luciferase as a reporter gene (Fig. 1). Notably, no Cas9 expression was detected in HT1080 cells infected with GGAAprom>Cas9 or GGAAless>Cas9. However, as expected, Cas9 was detected in cells infected with a lentivirus carrying Cas9 under the control of the EFS promoter, a mild constitutive promoter, which is expected to drive constitutive Cas9 expression in any cell type. The striking differences observed between Ewing sarcoma cells and fibrosarcoma cells cannot be attributed to variations in transduction efficiency, as all the cells exhibited similar GFP marker expression levels (Supplementary Fig. S4).

Once this strategy was demonstrated to be suitable for the expression of Cas9 nuclease specifically in Ewing sarcoma cells, we analyzed its ability to genetically inactivate EWSR1:FLI1 in these cells. For these experiments, we take advantage of our previous work in which we identified a combination of Cas9 and sgRNAs that was able to efficiently inactivate EWSR1 : : FLI1 [17]. As we showed previously, sgRNA directed against FLI1 exon 2 can be used as a control sgRNA in Ewing sarcoma cells because this FLI1 exon is not included in the EWSR1 : : FLI1 fusion, and Ewing sarcoma cells do not express native FLI1; thus, editing at this exon is not deleterious for Ewing sarcoma cells. In contrast, an RNA guide targeting FLI1 exon 9 affects the DNA-binding domain of FLI1, which is an essential region for EWSR1 : : FLI1 function.

Next, we infected GGAAprom>Cas9 and GGAAless>Cas9 cells with lentiviruses containing sgRNAs directed against FLI1 exon 2 (control) or FLI1 exon 9 and evaluated their effects on cell proliferation. As shown in Fig. 2B, the infection of GGAAprom A673 and MHH-ES1 cells with the FLI1-EX9 gRNA lentivirus reduced cell proliferation by over 95% compared with that of control cells (GGAAless cells). In contrast, no effects on cell proliferation were observed when GGAAprom and GGAAless cells were infected with lentivirus expressing the FLI1-EX2 gRNA, confirming that gene editing at this region of FLI1 is not deleterious to Ewing sarcoma cells [17]. As expected, no effects on cell proliferation were observed when GGAAprom>Cas9, GGAAless>Cas9, or EFS>Cas9 HT1080 cells were infected with FLI1 exon 2 or exon 9 gRNAs. These results demonstrate that the combination of Cas9 under the control of an Ewing-specific promoter (GGAAprom) and a gRNA targeting exon 9 of FLI1 is sufficiently efficient to block cell proliferation in Ewing sarcoma cells.

However, while lentiviral vectors are excellent proof-of-concept tools, the use of a lentiviral-based approach to deliver gene therapy to Ewing sarcoma cells is not suitable for clinical development. Consequently, we decided to explore the use of adenovirus as a suitable alternative to deliver gene therapy to Ewing sarcoma cells. Although adenoviruses have several drawbacks, mainly related to their immunogenicity, they remain one of the most commonly used viral vectors in cancer gene therapy, particularly for localized treatments.

First, we analyzed whether Ewing sarcoma cells can be efficiently infected with adenovirus. We infected Ewing sarcoma A673, MHH-ES1 and A4573 cells at different MOIs (25, 100, 400) with non-replicative adenovirus (serotype 5) carrying two different transgenes: mCherry under the control of a constitutive promoter and a luciferase gene under the control of either GGAAprom or the constitutive promoter EF1A. In addition to Ewing sarcoma cells, we also infected the fibrosarcoma cell line HT1080 and the osteosarcoma cell lines U2-OS and Saos-2. Quantification of mCherry-positive cells by flow cytometry demonstrated efficient transduction across all cell lines with both adenoviruses (Supplementary Fig. S5), with higher transduction efficiencies observed at higher MOI, as expected.

**Fig. 3:**
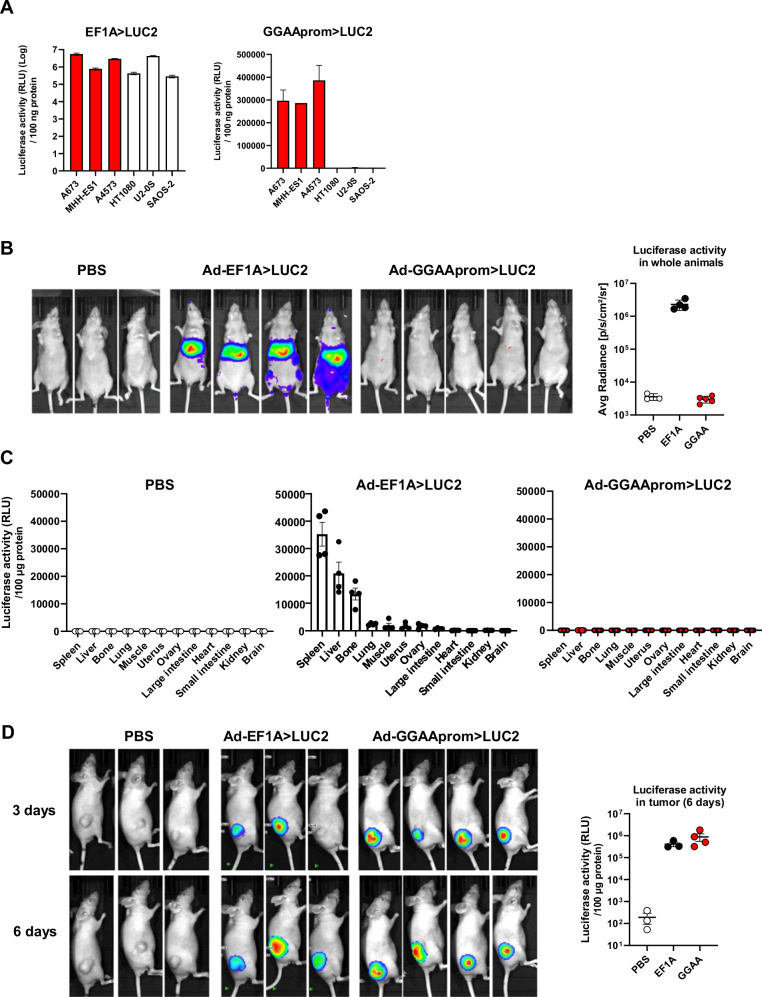
Exquisite specificity of luciferase gene expression under the control of a GGAAprom following adenovirus infection in vitro and in vivo. **A** Luciferase activity in cells infected with adenovirus (MOI = 100) harboring a constitutive promoter (EF1A > LUC2) or the Ewing specific promoter (GGAAprom>LUC2). GGAAprom was only active in Ewing sarcoma cells (red bars) (mean ± SD of a representative experiment from a total of three). **B** In vivo biodistribution experiments. Nude mice were injected i.v. with PBS (*n* = 3), Ad EF1A > LUC2 (*n* = 4) or Ad GGAAprom>LUC2, and luciferase activity quantified by IVIS imaging 72 hours post-injection. No luciferase activity was observed at all in mice injected i.v. with Ad GGAAprom>LUC2. **C** Quantification of luciferase activity in tissue extracts. Twelve different tissues were extracted from each animal and the corresponding protein lysates were analyzed for luciferase activity. Each point represents the luciferase activity obtained in one animal (mean ± SEM). **D** Luciferase expression in tumors injected i.t. with PBS (*n* = 3), Ad-EF1A > LUC2 (*n* = 3), and Ad-GGAAprom>LUC2 (*n* = 4). Luciferase activity was detected in Ewing sarcoma tumors injected with Ad-EF1A > LUC2 (constitutive expression) and Ad-GGAAprom>LUC2 (Ewing-specific expression). Luciferase activity in tumor tissue lysates is also shown. Each point represents the luciferase activity obtained in one tumor (mean ± SEM).

Next, we quantified the luciferase activity in cell lines infected at a MOI of 100, which resulted in transduction efficiencies >90% in all the cell lines with minimal toxicity (data not shown) (Fig. 3A). As expected, luciferase activity was detected in all cells infected with adenovirus carrying the luciferase gene under the control of the constitutive promoter EF1A. Notably, luciferase activity was also detected at very high levels in Ewing sarcoma cells expressing the luciferase gene under the Ewing-specific promoter GGAAprom, but not in the non-Ewing cell lines HT1080, Saos-2 and U2-OS, where luciferase levels were near background.

To further elucidate the cell/tissue specificity of a potential gene therapy based on adenovirus and GGAAprom, we intravenously injected nude mice with Ad-GGAAprom>LUC2 or Ad-EF1A > LUC2 and analyzed the distribution of luciferase activity in whole animals. As expected, animals injected with Ad-EF1A > LUC2 exhibited high levels of luciferase in the liver and spleen, as detected by in vivo imaging (Fig. 3B). Interestingly, no luciferase activity was detected by in vivo imaging in animals injected with Ad-GGAAprom>LUC2 (Fig. 3B). To increase the sensitivity of this experiment, we quantified luciferase activity in tissue lysates obtained from various organs. As shown in Fig. 3C, luciferase activity in nude mice injected with Ad-EF1A > LUC2 was primarily observed in the spleen, liver, and, strikingly, in bone. This indicates that, although adenoviruses are mainly sequestered by the liver and spleen, they can reach other tissues. In contrast, the levels of luciferase activity in tissue lysates from animals injected with Ad-GGAA > LUC2 were negligible across all tissues, including the liver and spleen (Fig. 3C). These findings strongly support that gene therapy based on the expression of a therapeutic gene under the control of the GGAAprom could represent a therapeutic strategy with remarkable specificity for Ewing sarcoma cells, without impacting normal cells of the organism.

After confirming the absence of off-target effects of Ad-GGAAprom>LUC2 in whole animals, we investigated whether adenovirus could effectively infect Ewing sarcoma cells in a conventional xenograft animal model. Nude mice were injected subcutaneously with A673 Ewing sarcoma cells, and when the tumors reached a mean volume of 250 mm^3^, were injected intratumorally with Ad-GGAAprom>LUC2 or Ad-EF1A > LUC2. As shown in Fig. 3D, tumors injected with either Ad-EF1A > LUC2 or Ad-GGAAprom>LUC2 exhibited high levels of luciferase activity, both in vivo and in tumor lysates. These results indicate that Ad-GGAAprom>LUC2 is able to infect Ewing sarcoma cells in vivo and efficiently induce the expression of a gene of interest upon reaching the target tissue, in this case, Ewing sarcoma cells.

**Fig. 4:**
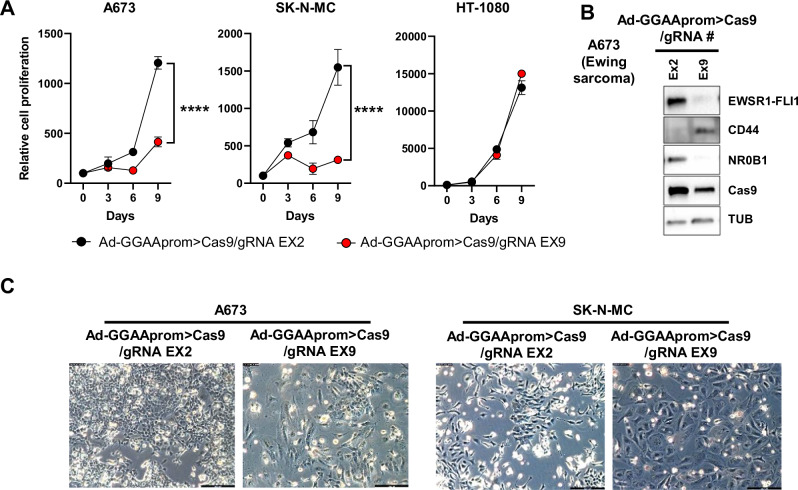
Inactivation of EWSR1 : : FLI1 in Ewing sarcoma cells following adenovirus infection. **A** Effect of Ad-GGAAprom>Cas9/gRNA EX2 and EX9 on cell proliferation. A673, SK-N-MC and HT-1080 cells were infected with the mentioned adenovirus (MOI = 25), and cell proliferation was monitored by cell counting. Cell proliferation was severely impaired in A673 and SK-N-MC Ewing sarcoma cells infected with Ad-GGAAprom>Cas9/gRNA EX9 (mean ± SD of a representative experiment from a total of three, ****P < 0.0001, Student’s T-test). **B** Expression of Cas9, EWSR1 : : FLI1, and EWSR1 : : FLI1 target genes CD44 and NR0B1 following adenovirus infection (MOI = 100). Treatment with Ad-GGAAprom>Cas9/gRNA EX9 dramatically reduced EWSR1 : : FLI1 expression, concomitantly affecting the expression of EWSR1 : : FLI1 target genes CD44 and NR0B1. **C** Effect of adenovirus infection on cell morphology. Flattened and rounded cells, characteristic of senescent cells, were predominant at day 9 post-infection.

Next, we analyzed the therapeutic efficacy of an adenovirus harboring Cas9 gene under the control of GGAAprom and FLI1 exon 9 gRNA under the control of the U6 promoter (Ad-GGAAprom>Cas9/gRNA EX9). Adenoviruses harboring the FLI1 exon 2 gRNA (Ad-GGAAprom>Cas9/gRNA EX2) were used as a negative control. Ewing sarcoma cell lines A673 and SK-N-MC and the fibrosarcoma cell line HT1080 were infected with these adenoviruses, and their effects on cell proliferation were assessed. As shown in Fig. 4A, Ad-GGAAprom>Cas9/gRNA EX9 dramatically affected the proliferation of the Ewing sarcoma cell lines A673 and SK-N-MC compared to cells infected with the control adenovirus Ad-GGAAprom>Cas9/gRNA EX2. Notably, the proliferation of the fibrosarcoma cell line HT1080 was not affected by Ad-GGAAprom>Cas9/gRNA EX9.

Next, we analyzed the effects of Ad-GGAAprom>Cas9/gRNA EX9 infection on EWSR1 : : FLI1 protein levels and two EWSR1 : : FLI1 target genes, CD44 [25] and NR0B1 [21], which serve as surrogate markers of EWSR1 : : FLI1 activity in A673 cells. As shown in Fig. 4B, Ad-GGAAprom>Cas9/gRNA EX9 caused a dramatic reduction in the level of EWSR1 : : FLI1 compared to control cells infected with Ad-GGAAprom>Cas9/gRNA EX2. This reduction in EWSR1 : : FLI1 protein levels correlated with a marked increase in CD44 protein levels (a gene negatively regulated by EWSR1 : : FLI1) and a strong decrease in NR0B1 protein levels (a gene positively regulated by EWSR1 : : FLI1). Cas9 expression was elevated in cells infected with both adenoviruses. Consistent with this finding, more than 30% of indels in exons 2 and 9 of FLI1 were detected on day 3 post-infection (Supplementary Fig. S6).

The effect of genetic inactivation of EWSR1 : : FLI1 on cell morphology was also evident. Thus, large cells with a flattened and rounded morphology, characteristic of senescent cells, were first observed on day 6 post-infection and became predominant on day 9 (Fig. 4C). These findings are consistent with our previously published results [17] and confirm the negative impact of Ad-GGAAprom>Cas9/gRNA EX9 on the proliferation of Ewing sarcoma cells.

**Fig. 5:**
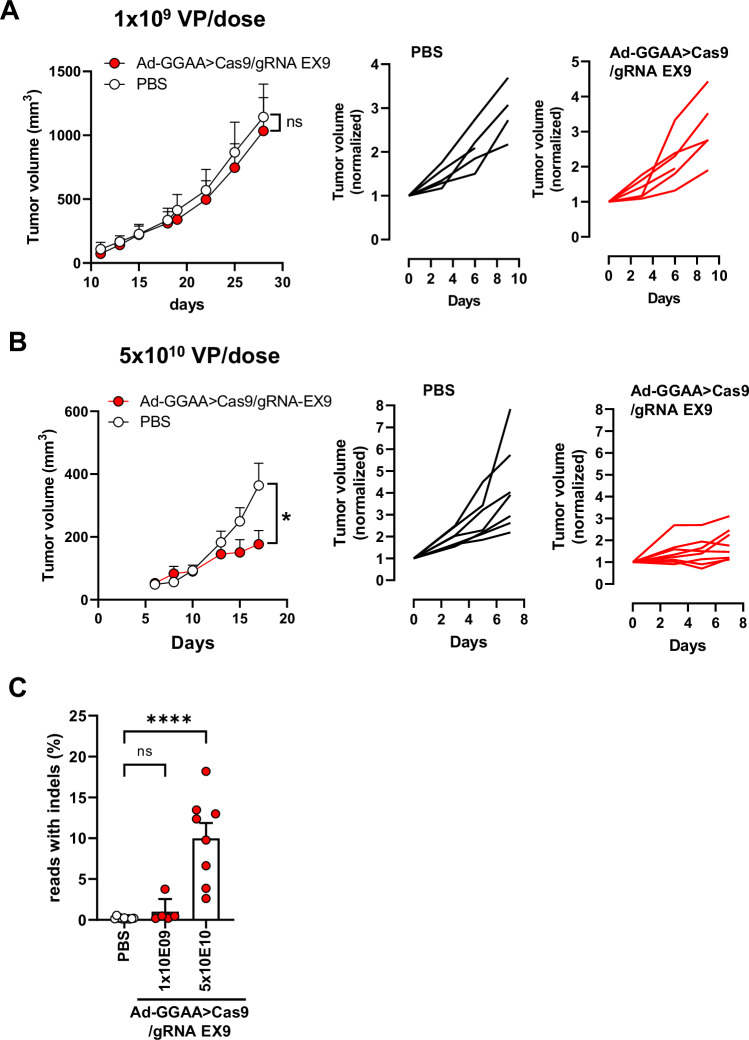
Intratumoral treatment with Ad-GGAAprom>Cas9/gRNA EX9 reduces tumor growth in vivo. **A** Nude mice were inoculated s.c. with A673 cells and split into two groups on day 19. The group treated with adenovirus received intratumoral injections of Ad-GGAA>Cas9/gRNA EX9 at days 19, 22 and 25 (1 × 10^9^ VP/dose) (*n* = 5). Control group received intratumoral PBS (*n* = 5). Tumor volume and tumor volume normalized to day 19 (start of treatment) for each animal are showed. **B** In the second experiment, adenovirus treatment (5 × 10^10^ VP/dose) was started on day 9 *(n* = 8) and administered at days 10, 13 and 15. Control group received intratumoral PBS as above (*n* = 7). Tumor volume and tumor volume normalized to day 9 (start of treatment) for each animal are showed. A significant reduction in tumor growth was observed in the treated group (*P < 0.05; two-way ANOVA). **C**) At the end of the experiment, tumors were excised, and gene editing was quantified by ultra-deep next generation sequencing. Significant gene editing was observed in animals treated with 5 × 10^10^ VP/dose (mean ± SEM; ****P < 0.0001, one-way ANOVA).

Finally, we analyzed the effect of the intratumoral administration of Ad-GGAAprom>Cas9/sgRNA-FLI1-EX9 on the growth of A673 tumor xenografts. Athymic nude mice were injected subcutaneously with A673 cells, and 19 days later, tumors were treated intratumorally with Ad-GGAAprom>Cas9/gRNA EX9 (1 × 10^9^ VP/100 µL per dose) or PBS on days 19, 22 and 25. As shown in Fig. 5A, no significant effects on tumor growth were observed in the group treated with adenovirus compared with the control group. Since in vitro studies demonstrated a significant effect of adenovirus treatment on cell proliferation, we reasoned that this lack of effectiveness in vivo might be due to insufficient dosage. To evaluate this, we performed a new in vivo experiment where tumors were treated intratumorally on days 10, 13 and 15 with a dose of 5 x 10^10^ VP/dose (Fig. 5B). At this dose, the reduction in tumor growth rate was significant and maintained until the end of the experiment. When tumor volume was normalized to the tumor volume on the day of treatment initiation, it was evident that most tumors experienced no tumor growth or grew only very slightly (Fig. 5B).

Consistent with the effects on tumor growth, gene editing rates were very low or near-background in the tumors treated with 1 × 10^9^ VP/dose (Fig. 5C and Supplementary Fig. S7). However, gene editing rates ranged from 3 to 18% in tumors treated with 5 × 10^10^ VP/dose, demonstrating a positive correlation between EWSR1 : : FLI1 inactivation and tumor growth (Fig. 5C and Supplementary Fig. S7).

**Fig. 6:**
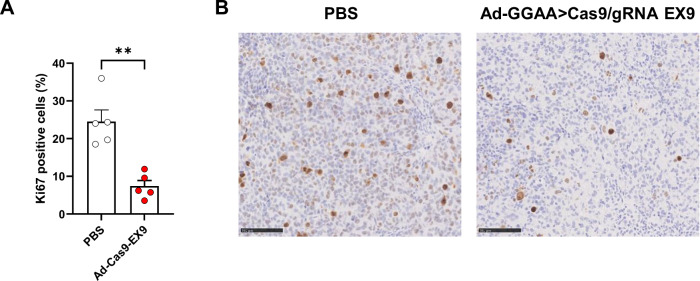
Intratumoral treatment with Ad-GGAAprom>Cas9/gRNA EX9 reduces the levels of Ki67+ cells. **A** Tumor sections were stained with an anti-Ki67 antibody to assess cell proliferation. Graph shows percentage of Ki67+ cells in control animals (PBS, *n* = 5) and animals treated with 5 × 10^10^ VP/dose of Ad-GGAAprom>Cas9/gRNA EX9 (Ad-Cas9-EX9, *n* = 5) (mean ± SEM; **P < 0.001, Student’s *T*-Test). **B** Representative tumors for each group stained with Ki67 antibody (bar = 100 µm).

Finally, we performed immunohistochemistry staining with an anti-Ki67 antibody (a clinically validated marker of cell proliferation) on tissue tumor sections to confirm the effects of adenovirus treatment at 5 × 10^10^ VP/dose on tumor cell proliferation. As shown in Fig. 6, there was a significative reduction in the percentage of Ki67 positive cells in tumors treated with adenovirus when compared to the control group.

## Discussion

Ewing sarcoma is a devastating rare tumor affecting children and young adults, for which the development of new therapeutic strategies is imperative. From a molecular point of view, Ewing sarcoma is characterized by tumor-specific chromosomal translocations that produce tumor-specific fusion proteins, such as the oncogenic chimeric transcription factor EWSR1 : : FLI1. Interestingly, Ewing sarcoma cells are addicted to these EWSR1 : : FLI1 fusion oncoproteins; thus, any approach aimed at blocking or inactivating EWSR1 : : FLI1 is expected to interfere with tumor growth.

With this objective in mind, several research groups have focused on identifying small molecules that are able to interfere with the transcriptional activity of EWSR1 : : FLI1 fusion proteins. Some of these small molecules include trabectedin and its analogs [26, 27], mithramycin [28, 29], and YK-4-279 and its derivatives (for example, TK216) [30, 31]. Although these small molecules initially seemed to offer an exciting alternative for treating patients with Ewing sarcoma, the results of clinical trials have been disappointing [32, 33]. Furthermore, using small molecules to interfere with the activity of EWSR1 : : FLI1 probably would require continuous drug administration to obtain a long-lasting response, which could ultimately lead to unwanted resistance, as observed with tyrosine kinase inhibitors (for example, imatinib) [34], thereby limiting the effectiveness of this strategy [35].

Another possible strategy to interfere with EWSR1 : : FLI1 function is the use of siRNAs to knock down EWSR1 : : FLI1 expression. This strategy has been extraordinarily successful for the generation of cell models of EWSR1 : : FLI1 knockdown, which have been very helpful for understanding the biology of Ewing sarcoma. However, potential therapies based on the use of siRNAs designed to knockdown EWSR1 : : FLI1 mRNAs have the same drawbacks as those described above concerning the use of small molecules designed to block EWSR1 : : FLI1 function. Therefore, to achieve the best EWSR1 : : FLI1 inactivation, it would be desirable to obtain complete, that is, genetic inactivation of the *EWSR1 :* *: FLI1* gene.

We have recently shown that EWSR1 : : FLI1 genetic inactivation is possible using CRISPR/Cas9 technologies and that EWSR1 : : FLI1 genetic inactivation blocks Ewing sarcoma cell proliferation [17]. However, as we pointed out in our previous work, exquisite control of Cas9 expression must be achieved to rule out any unwanted effects on normal cells. In the present work, we demonstrate that this precise and exquisite control could be achieved by using an Ewing-specific promoter formed by concatenated repeats of the tetranucleotide GGAA (GGAAprom). Interestingly, the use of a consensus TATA box downstream of the GGAA repeats increased the activity of the promoter by 2–3 times compared to that of the GGAA repeats only, without affecting their specificity. The high specificity of this DNA sequence for Ewing sarcoma cells was demonstrated both in vitro and in vivo. Thus, GGAAprom was active in Ewing sarcoma cells expressing EWSR1 : : FLI1 proteins (A673, MHH-ES1, A4573, which encompass three different EWSR1 : : FLI1 isoforms) but not in non-Ewing sarcoma cells such as the fibrosarcoma cell line HT1080 or the osteosarcoma cell lines U2-OS and Saos-2. The absence of GGAAprom activation in HT1080 cells is particularly relevant because these cells express high levels of native FLI1, indicating that GGAAprom is only active in Ewing sarcoma cells expressing EWSR1 : : FLI1. This important finding was soundly confirmed in in vivo experiments. Thus, when athymic nude mice were injected intravenously with adenovirus harboring a luciferase gene under the control of GGAAprom, no luciferase expression in any of the 12 tissues analyzed was detected. This means that a gene therapy in which the therapeutic gene is under the control of the Ewing-specific promoter will be active only in Ewing sarcoma cells expressing EWSR1 : : FLI1, ruling out possible off-target effects. This opens the possibility of using this type of approach with a multitude of therapeutic genes that may include not only nucleases such as the one proposed in this work to inactivate EWSR1 : : FLI1 but also genes capable of inducing cell death (for example, suicide genes) or immunomodulatory genes capable of activating an antitumor immune response.

Although the gene therapy proposed here is effective in permanently inactivating EWSR1 : : FLI1, several aspects should be addressed and taken into account before moving towards preclinical and clinical development. First, the in vivo experiments performed clearly demonstrate that one of these aspects is the dose of adenovirus administered. Thus, while a dose of 1 x 10^9^ VP/dose clearly had no effect on tumor growth, a 50-fold higher dose of 5 × 10^10^ VP/dose blocked tumor growth for the duration of treatment. This blockage of tumor growth was associated with a significant decrease in tumor cell proliferation rates. It is noteworthy that the highest dose used in our in vivo studies remains significantly lower than those used in clinical trials with other therapeutic adenoviruses. For example, in several clinical trials evaluating the therapeutic potential of intratumoral administration of a non-replicating adenovirus harboring the HSV-TK suicide gene, the adenovirus dose ranged from 1 x 10^11^ to 1 x 10^12^ VP/dose [36–38], still one or two orders of magnitude higher than that used in our preclinical animal studies. This indicates that there is still room to use higher doses that would have yielded even more remarkable results. Unfortunately, further experiments with higher doses were not possible due to the limitation in the amount of adenovirus produced.

As a proof of concept, we opted for intratumoral administration of adenovirus, which is, as noted above, a route frequently used in clinical trials to deliver virus-based therapies in cancer [36–38]. However, if it were possible, the route of administration should ideally be intravenous in order to reach any tumor mass. In this sense, our results have clearly demonstrated that intravenous administration of adenovirus harboring a luciferase gene under the control of GGAAprom is safe, with no expression of the reporter gene in any normal tissue. However, although expression of the reporter gene (or, where appropriate, the therapeutic gene) does not occur in the liver or spleen, eliminating the possibility of relevant off-target effects, yet adenoviruses will be rapidly cleared by the liver, thus reducing the amount of adenovirus available to reach the tumor. In this context, adenovirus research is continuously evolving, and new and advanced adenovirus variants have been designed to reduce clearance in the liver and decrease the intrinsic immunogenicity of the adenovirus [39].

One of the main limitations of the strategy outlined in this work is that gene therapy must theoretically reach all tumor cells to be truly effective, since those cells that escape inactivation of the *EWSR1 :* *: FLI1* gene will be able to continue proliferating and maintain the growth of the tumor. However, it is also possible that the death of a certain percentage of tumor cells may in turn activate a specific tumor immune response, such as the release of neoantigens released by dead tumor cells. Additionally, the expression of Cas9 by tumor cells is itself a source of neoantigens that have the capacity to activate the immune system [40, 41]. Ewing sarcoma belongs to a group of tumors classified as “cold tumors” in which immunotherapies have proven to be ineffective [42]. As has been recently shown, this seems to be due to the repressive effect that Ewing sarcoma cells maintain on the immune system itself, limiting the capacity for infiltration and activation of immune effector cells [43, 44]. It will be very interesting to confirm whether genetic inactivation of EWSR1 : : FLI1 in at least a subset of Ewing sarcoma cells is able to activate a tumor-specific response of the immune system in humanized mouse models [45, 46].

Recently, Hölting and coworkers [47] published a similar strategy to the one described by us in this work, to express the suicide gene thymidine kinase (HSV-TK) specifically in Ewing sarcoma cells by using a synthetic GGAA promoter. While the findings of Hölting and our own clearly support the usefulness of this strategy to express therapeutic genes specifically in Ewing sarcoma, our work includes some new interesting findings that are now reported for the first time. First, we describe a complete study regarding the in vivo specificity of the therapy, demonstrating that no off-target expression can be observed in mice when adenovirus is administered intravenously. This is a key aspect of our approach, which justifies continuing its preclinical development. Second, in our work, we tested the use of adenoviruses as putative vectors to deliver gene therapy and demonstrated that adenoviruses can infect Ewing sarcoma cells and transduce them both in vitro and in vivo. Despite some of the limitations of adenoviruses, they are well-established vectors for gene therapy in cancer, with dozens of clinical trials carried out and some adenovirus-based therapies approved on the market [48, 49]. In contrast, in the aforementioned article, the authors use a complex strategy for in vivo delivery based on the use of lentiviral vectors and an adapter antibody that, in our opinion, can make more difficult its preclinical development.

The field of gene therapy based on the use of CRIPSR/Cas9 to correct/inactivate genes and thus treat somatic diseases with a genetic basis is a rapidly evolving field. In this continuous advance, one of the diseases that can most quickly benefit from these technologies is undoubtedly cancer, but also other diseases (hypertension, diabetes, etc.), not only from a therapeutic point of view, but also in relation to diagnosis or the generation of more specific models of the disease [50–52]. Although in vivo gene editing certainly presents numerous risks that must be carefully considered, the development of highly specific expression strategies such as the one presented in this work opens new opportunities to permanently inactivate the EWSR1 : : FLI1 fusion gene characteristic of Ewing sarcoma.

In summary, in this work, we reported a gene therapy approach to permanently inactivate *EWSR1 :* *: FLI1* in Ewing sarcoma cells in a highly specific way. To our knowledge, no other approach has been shown to inactivate so effectively, and particularly, so specifically, the *EWSR1 :* *: FLI1* gene. We believe the results shown here support the use of this strategy to treat Ewing sarcoma and justify continuing its preclinical development.

## Supplementary information

Supplementary Figures S1-S7

## Data Availability

The data presented in this study are available from the corresponding author on reasonable request.
