# Supplementary material for: Targeted inactivation of EWSR1 : : FLI1 gene in Ewing sarcoma via CRISPR/Cas9 driven by an Ewing-specific GGAA promoter
Source: Cancer Gene Ther. 2025 Mar 15;32(4):437–49. doi: 10.1038/s41417-025-00887-8 (PMC11976297; doi:10.1038/s41417-025-00887-8)

Supplementary Figure S1

(Cervera, S.T. et al.)

Supplementary Figure S1. Sequence of GGAAprom. DNA sequence cloned in pGL3basic reporter vector. GGAA-microsatellite from NR0B1 promoter (Ewing sarcoma A4573 cells) was PCR-amplified, cloned in pGEM-Teasy vector, and afterward cloned in the KpnI/Sac1 sites of pGL3basic. GGAA repeats (x 25) are underlined and the TATA box generated during cloning is shown in bold type.

GGTACCTCTCACAGGCAGAATGAAATTTAACGCTGCAAGCAAAATGGGGGTCTCCTAGGTTTTC
CTCTTATGCTGAGAATTCCAGGTCCTGGAGAAGAAGAAAAAGAGAAAGAAAGAGAGAGAGAAGG
AGTGAGAGAGGGAGGGAGGGAGGGAGGGAGGGAGGAAGGAAGGAAGGAAGGAAGGAAGGAAAGG
AAGGAAGGAAGGAAGGAAGGAAGGAAAGGAAGGAAGGAAGGAAGGAAGGAAGGAAGGAAGGAAG
GAAGGAAAAGAAACAGCAAAAAAAGAAAGAGGGAGGATGGGAGGGAGGGAAAAAGTAAAAATGA
TTCTGTATCAGCTGG**TATAAA**TCACTAGTGAATTCGCGGCCGCCTGCAGGTCGACCATATGGGA
GAGCTC

Supplementary Figure S2. Sequences of GGAAprom variants cloned in lentiviral plasmids. GGAA repeats are underlined. TATA box is showed in bold types. Sequences derived from the lentiviral backbone are showed in lowercase letters. ATG from luciferase gene is underlined.

GGAAprom from pLV[Exp]-EGFP:T2A:Bsd-{GGAAprom}>Luc2

aaaagttgCTCTCACAGGCAGAATGAAATTTAACGCTGCAAGCAAAATGGGGGTCTCCTAGGTT
TTCCTCTTATGCTGAGAATTCCAGGTCCTGGAGAAGAAGAAAAAGAGAAAGAAAGAGAGAGAGA
AGGAGTGAGAGAGGGAGGGAGGGAGGGAGGGAGGGAGGAAGGAAGGAAGGAAGGAAGGAAGGAA
AGGAAGGAAGGAAGGAAGGAAGGAAGGAAAGGAAGGAAGGAAGGAAGGAAGGAAGGAAGGAAGG
AAGGAAGGAAAAGAAACAGCAAAAAAAGAAAGAGGGAGGATGGGAGGGAGGGAAAAAGTAAAAA
TGATTCTGTATCAGCTGG**TATAAA**TCACTAGTGAATTCGCGGCCGCCTGCAGGTCGACCATATG
GGAGAGCTcaagtttgtacaaaaaagcaggctgccaccatg

GGAAprom TATAless from pLV[Exp]-EGFP:T2A:Bsd-{GGAAprom-TATAless}>Luc2

aaaagttgCTCTCACAGGCAGAATGAAATTTAACGCTGCAAGCAAAATGGGGGTCTCCTAGGTT
TTCCTCTTATGCTGAGAATTCCAGGTCCTGGAGAAGAAGAAAAAGAGAAAGAAAGAGAGAGAGA
AGGAGTGAGAGAGGGAGGGAGGGAGGGAGGGAGGGAGGAAGGAAGGAAGGAAGGAAGGAAGGAA
AGGAAGGAAGGAAGGAAGGAAGGAAGGAAAGGAAGGAAGGAAGGAAGGAAGGAAGGAAGGAAGG
AAGGAAGGAAAAGAAACAGCAAAAAAAGAAAGAGGGAGGATGGGAGGGAGGGAAAAAGTAAAAA
TGATTCTGTATCAGCTGGTCACTAGTGAATTCGCGGCCGCCTGCAGGTCGACCATATGGGAGAG
CTcaagtttgtacaaaaaagcaggctgccaccatg

GGAAprom GGAAless from pLV[Exp]-EGFP:T2A:Bsd-{GGAAprom GGAAless}>Luc2

aaaagttgCTCTCACAGGCAGAATGAAATTTAACGCTGCAAGCAAAATGGGGGTCTCCTAGGTT
TTCCTCTTATGCTGAGAATTCCAGGTCCTTAAAAATGATTCTGTATCAGCTGG**TATAAA**TCACT
AGTGAATTCGCGGCCGCCTGCAGGTCGACCATATGGGAGAGCTcaagtttgtacaaaaaagcag
gctgccaccatg

GGAAprom GGAAless TATAless from pLV[Exp]-EGFP:T2A:Bsd-{GGAAprom GGAAless TATAless}>Luc2

aaaagttgCTCTCACAGGCAGAATGAAATTTAACGCTGCAAGCAAAATGGGGGTCTCCTAGGTT
TTCCTCTTATGCTGAGAATTCCAGGTCCTTAAAAATGATTCTGTATCAGCTGGTCACTAGTGAA
TTCGCGGCCGCCTGCAGGTCGACCATATGGGAGAGCTcaagtttgtacaaaaaagcaggctgcc
accatg

Supplementary Figure S3
(Cervera, S.T. et al.)

Supplementary Figure S3. Transduction levels of GGAAprom-LUC2 lentiviral vectors in A673 cells (Ewing sarcoma) and HT1080 cells (Fibrosarcoma). A) Quantification of GFP positive cells by flow-cytometry in stably transduced cells (MOI=5). The percentage of transduced cells (>95%) was similar in all cases. **B)** Protein expression of EWSR1::FLI1 and FLI1 in stably transduced cells. Western-blots were hybridized with an antibody against the C-terminal region of FLI1 that recognizes consequently both EWSR1::FLI1 and native FLI1 proteins. A673 Ewing sarcoma cells express EWSR1::FLI1 proteins, but not native FLI1. HT1080 fibrosarcoma cells express native FLI1 protein, but not EWSR1::FLI1.

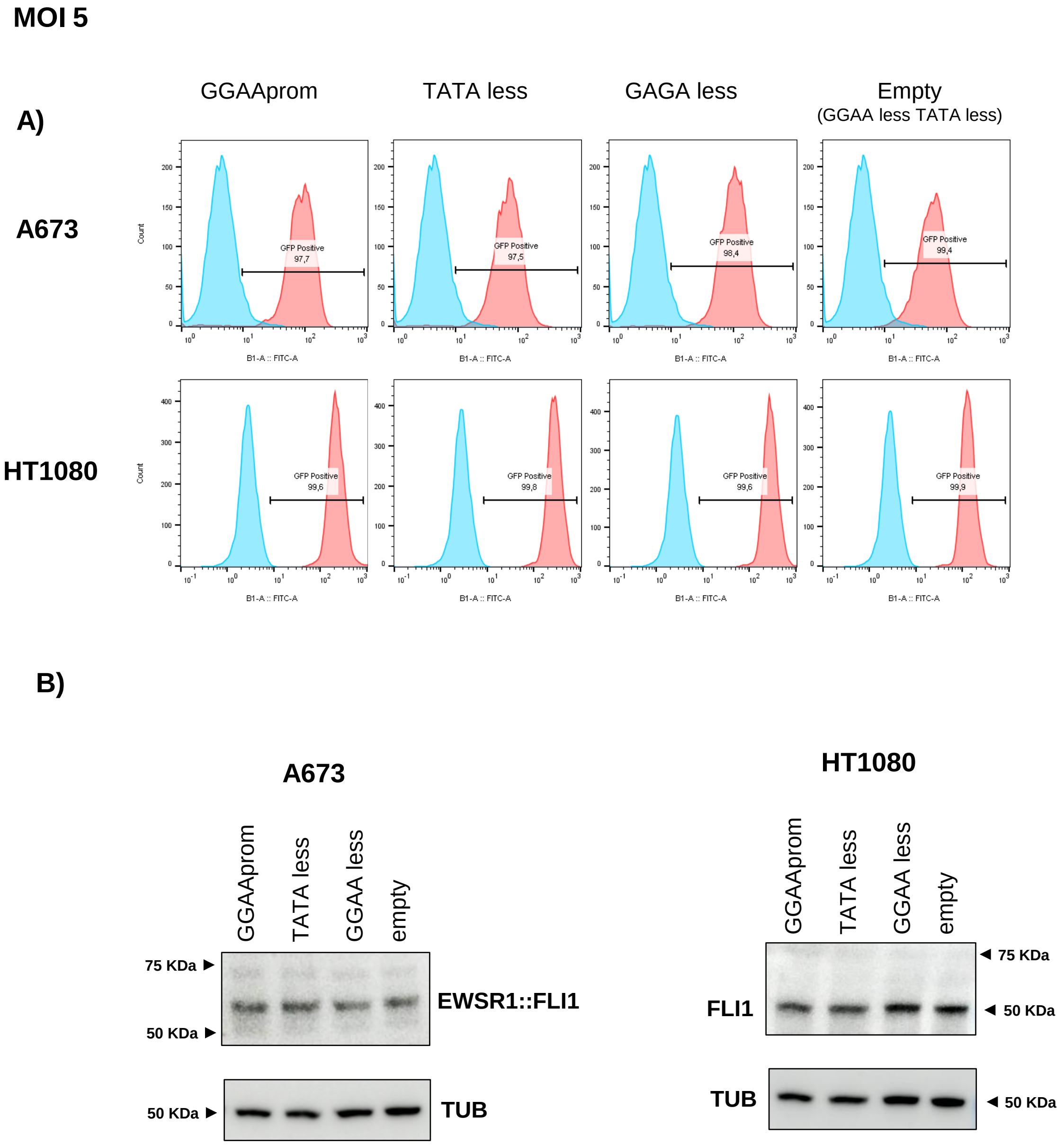

Supplementary Figure S3 (Continued)

(Cervera, S.T. et al.)

Supplementary Figure S3 (continued). C) Quantification of GFP positive cells by flow-citometry in stably transduced cells (MOI=0.5). **D)** Protein expression of EWSR1::FLI1 and FLI1 in stably transduced cells. A673 and MHH-ES1 Ewing sarcoma cells express EWSR1::FLI1 proteins, but not native FLI1. HT1080 fibrosarcoma cells express native FLI1 protein, but not EWSR1::FLI1.

MOI 0.5

C)

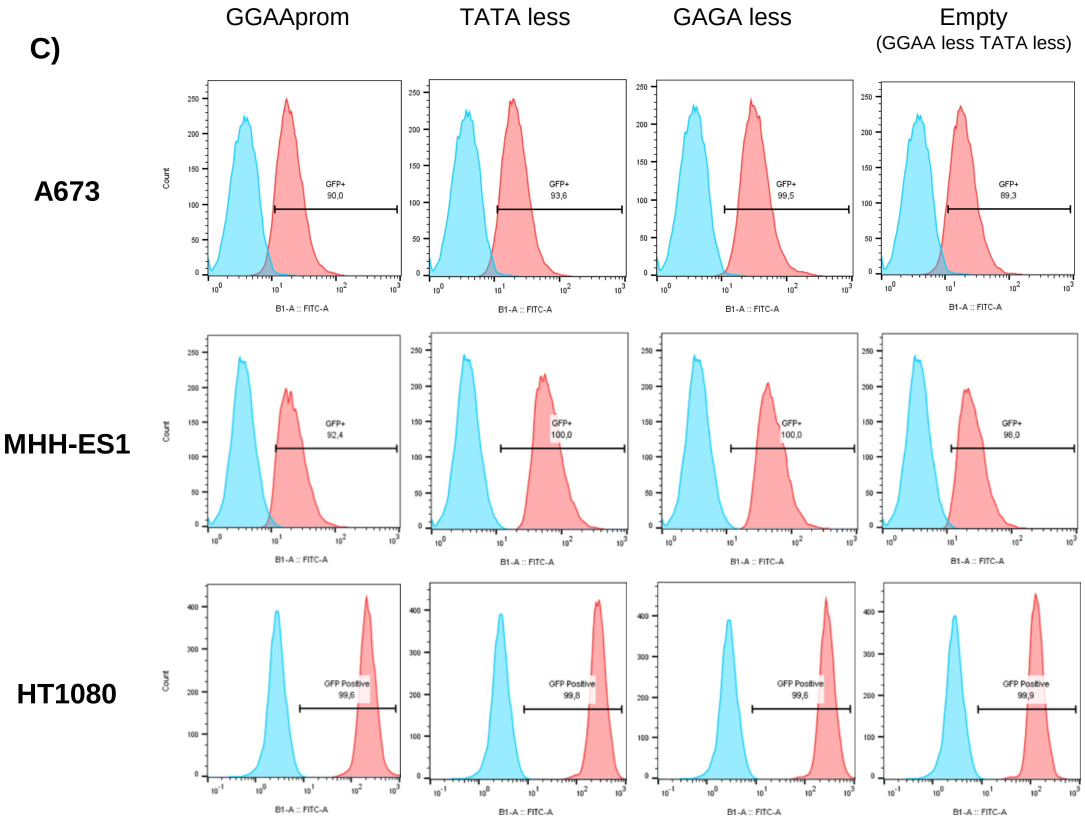

D)

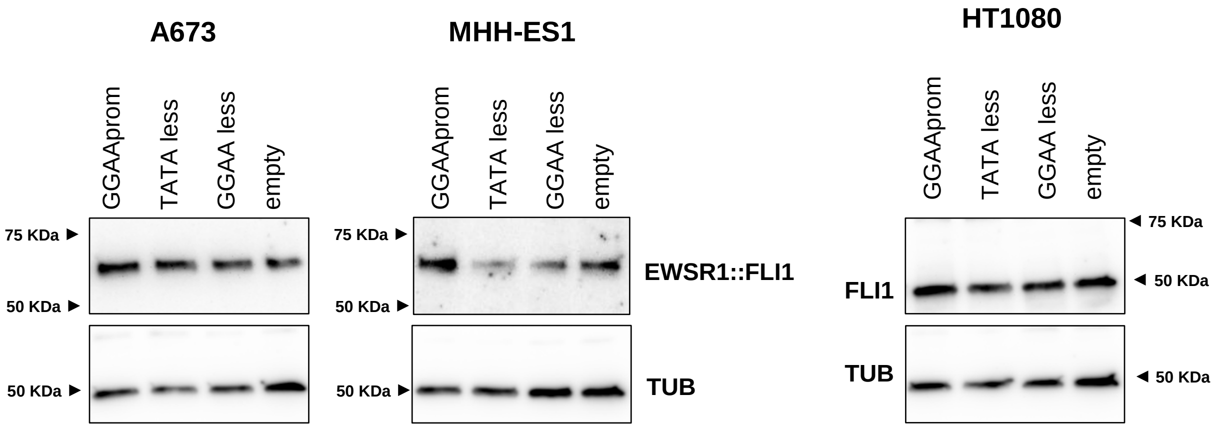

Supplementary Figure S4
(Cervera, S.T. et al.)

Supplementary Figure S4. Transduction levels of Cas9 lentiviral vectors in A673 and MHH-ES1 cells (Ewing sarcoma) and HT1080 cells (Fibrosarcoma). Quantification of GFP positive cells by flow-citometry in stably transduced cells. The percentage of transduced cells (>95%) was similar in all cases.

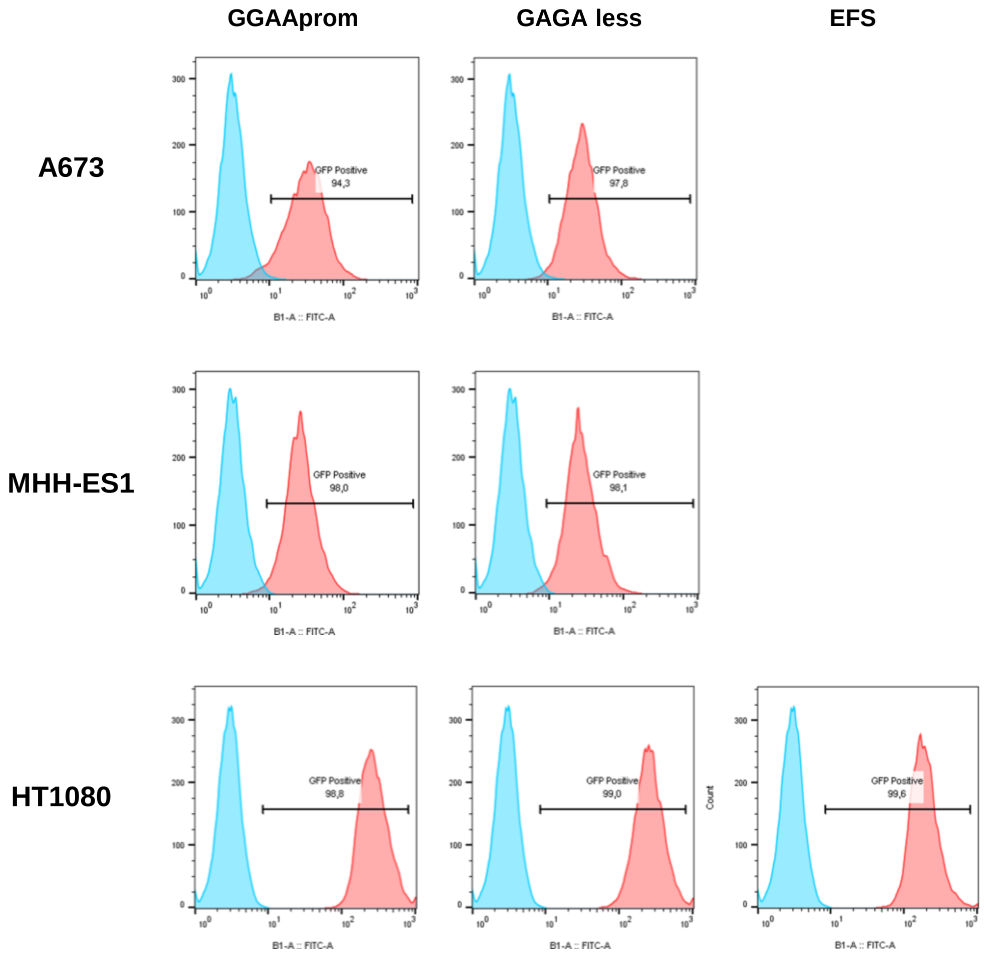

Supplementary Figure S5
(Cervera, S.T. et al.)

Supplementary Figure S5. Transduction levels in Ewing sarcoma cells (A673, MHH-ES1 and A4573), fibrosarcoma (HT1080) and osteosarcoma cells (U2-OS and SAOS-2) infected with adenovirus EF1A>LUC2 and GGAAprom>LUC2. Quantification of mCherry positive cells by flow-cytometry.

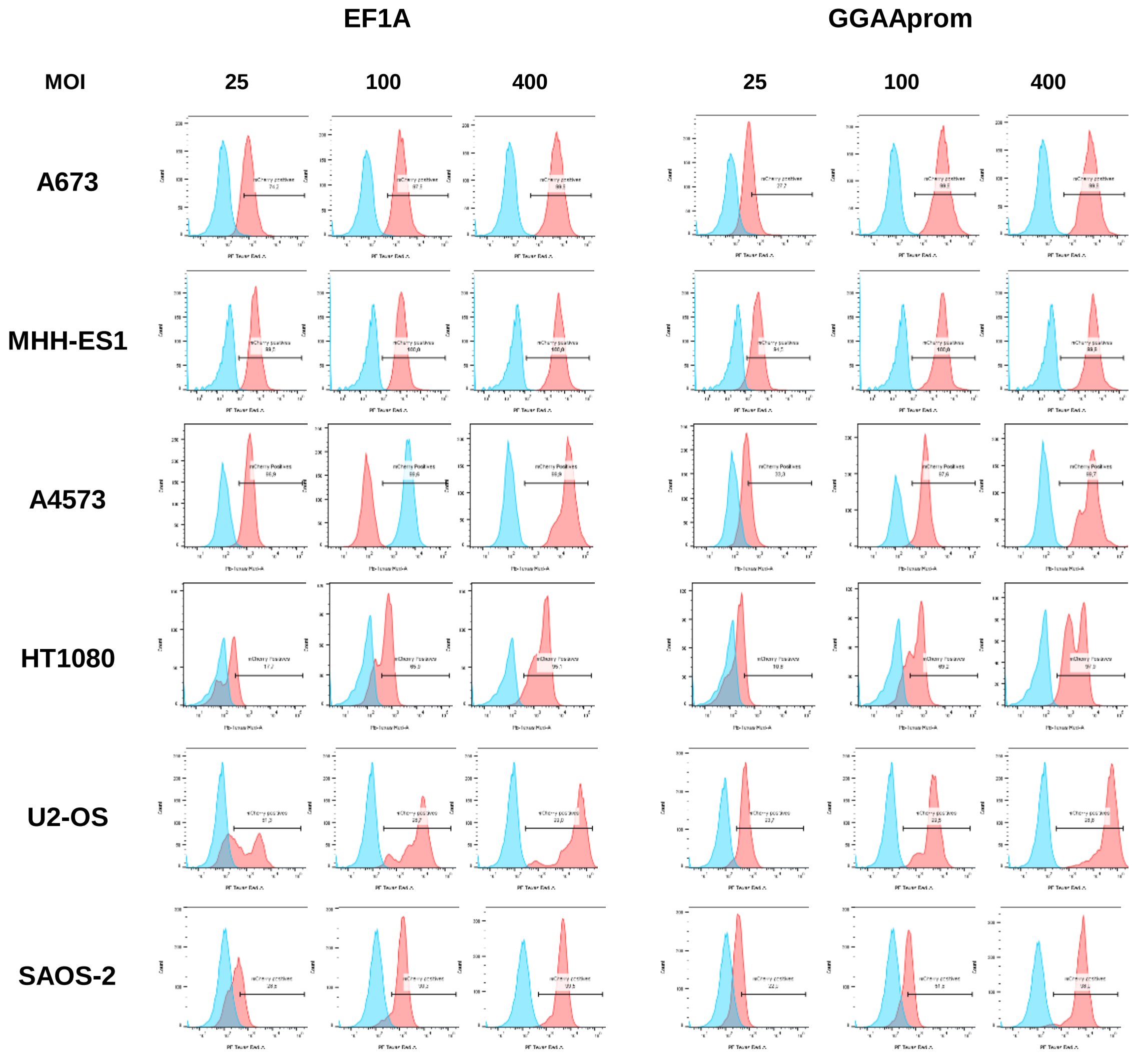

Supplementary Figure S6
(Cervera, S.T. et al.)

Supplementary Figure S6. Gene editing upon infection with adenovirus GGAA>Cas9. A673 cells were infected with Ad-GGAA>Cas9-U6>gRNA-FLI1-EX9 or Ad-GGAA>Cas9-U6>gRNA-FLI1-EX2 (negative control) and gene editing analyzed on day 3 post-infection. **A)** T7 endonuclease assay, showing percentage of altered PCR products. **B)** Electropherograms analyzed with ICE CRISPR analysis tool showing the percentage of indels detected. **C)** Primer sequences used in PCR.

A)

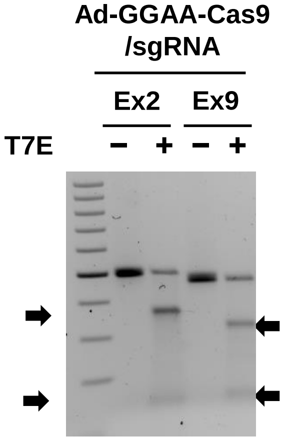

B)

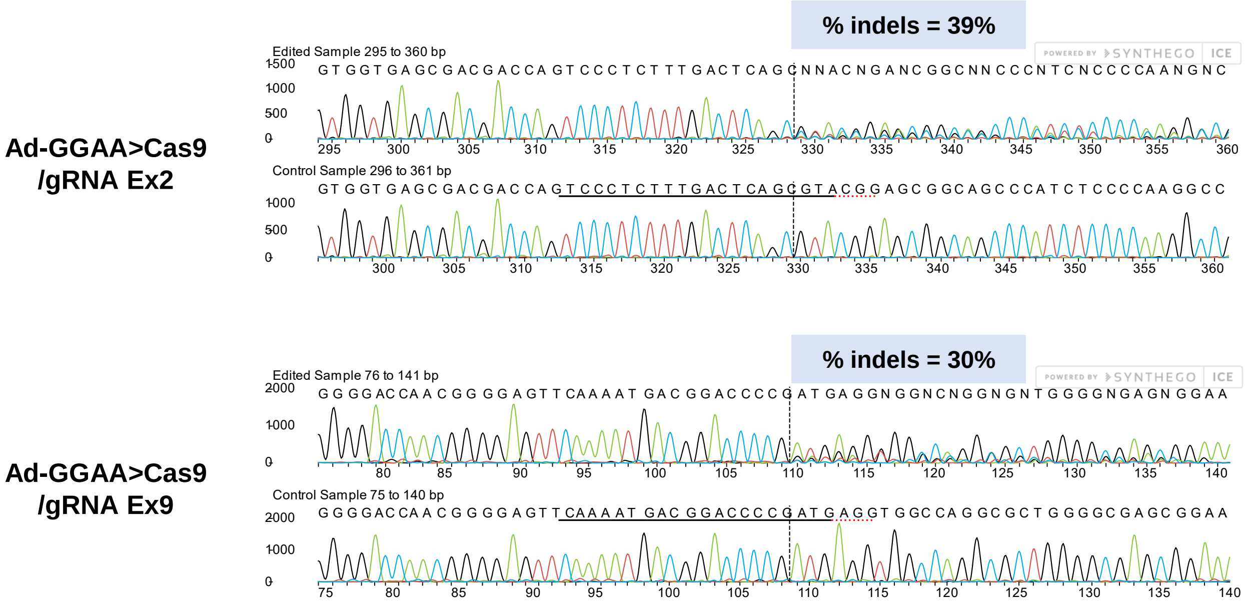

C)

Primer	Sequence
FLI1-EX2-F	5’-CCACTATTCTTGGCCTCCCT-3’
FLI1-EX2-R	5’-TTGACCCTCACTGGCTGATT-3’
FLI1-EX9-F	5’-TTCTCTCCCGTTTGCCTCAC-3’
FLI1-EX9-R	5’-TGTGATGCGGCTCCAAAGAA-3’

Supplementary Figure S7

(Cervera, S.T. et al.)

Supplementary Figure S7. Gene editing in *in vivo* models. Tumor DNA was isolated from tumors and then PCR-amplified with primers described in Figure S6 for FLI1 exon 9. Then, PCR amplicons were purified, PCR-indexed, repurified, quantified, and pooled. Sequencing was performed in a MiSeq sequencer using a 2x250 pb paired-end reads scheme. Percentage of edited reads was calculated with CRISPResso2 software (http://crispresso2.pinellolab.org/submission). Data from representative samples of each *in vivo* experiment and each experimental group (Ad-FLI1-EX9 and PBS) are showed.

Ad-GGAA>Cas9/
gRNA EX9 #1
Experiment 1
(1x10^{9} VP/dose)
(% edited reads = 3.75)

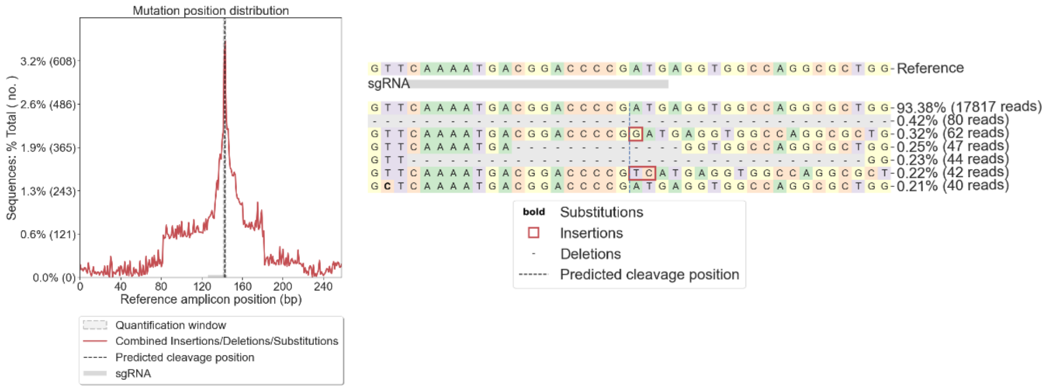

Ad-GGAA>Cas9/
gRNA EX9 #2
Experiment 2
(5x10^{10} VP/dose)
(% edited reads = 18.2)

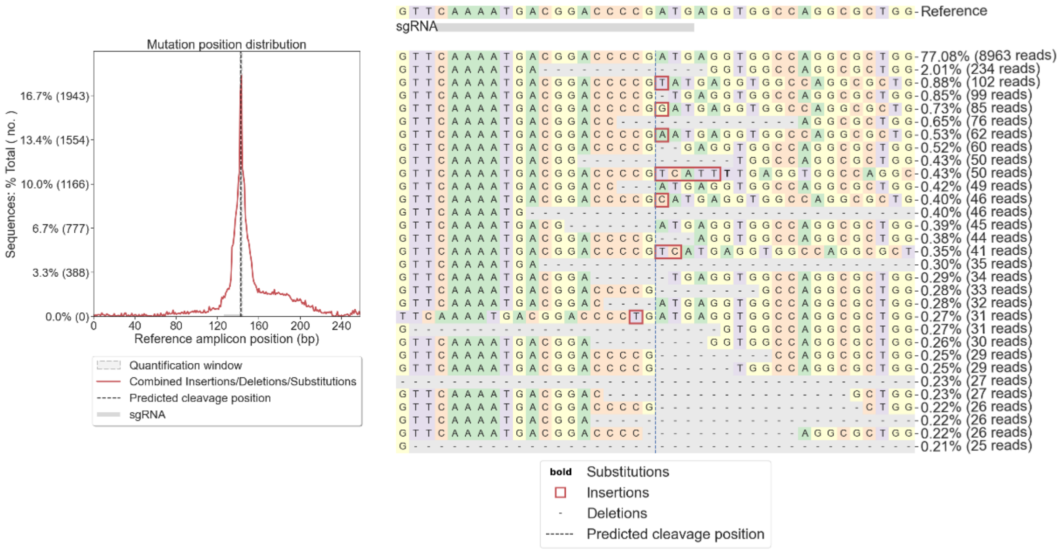

Supplementary Figure S7 (continued)

(Cervera, S.T. et al.)

PBS #13
Experiment 1
(% edited reads = 0.1)

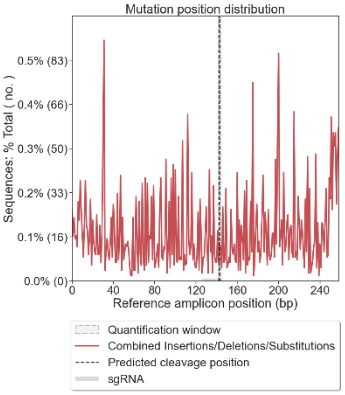

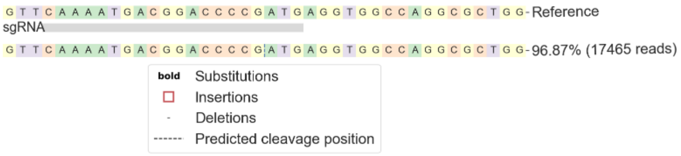

PBS #7
Experiment 2
(% edited reads = 0.1)

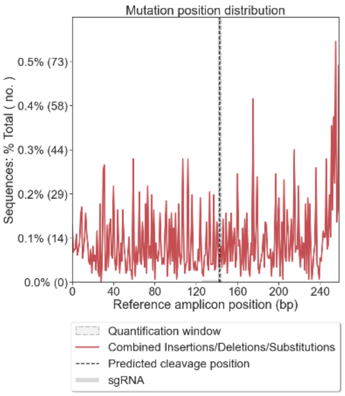

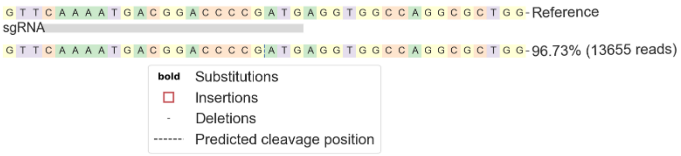

Supplement: Supplementary file 1 — Supplementary Figures S1-S7 [file 41417_2025_887_MOESM1_ESM.pdf]
